# Glioblastoma multiforme versus pleomorphic xanthoastrocytoma with anaplastic features in the pathological diagnosis: a case report

**DOI:** 10.1186/s13000-016-0514-2

**Published:** 2016-07-22

**Authors:** Shoko M. Yamada, Hideki Murakami, Yusuke Tomita, Makoto Nakane, Soichiro Shibui, Mikiko Takahashi, Masashi Kawamoto

**Affiliations:** Department of Neurosurgery, Teikyo University Mizonokuchi Hospital, 3-8-3 Mizonokuchi, Takatsu-ku, Kawasaki, Kanagawa 213-8507 Japan; Department of Diagnostic Pathology, Teikyo University Mizonokuchi Hospital, 3-8-3 Mizonokuchi, Takatsu-ku, Kawasaki, Kanagawa 213-8507 Japan

**Keywords:** Pleomorphic xanthoastrocytoma, Glioblastoma, Anaplastic features, Ki-67 index

## Abstract

**Background:**

Pleomorphic xanthoastrocytoma (PXA) with anaplastic features should be strictly distinguished from glioblastoma multiforme (GBM).

**Case presentation:**

A case of PXA that was initially diagnosed as GBM is presented. A 42-year-old man visited our clinic because of right hemiparesis and total aphasia. Head magnetic resonance imaging demonstrated enhanced multiple cystic lesions in the left temporal lobe suggesting an intra-parenchymal brain tumor. The lesion was partially removed and GBM with a Ki-67 index of 20 % was diagnosed by pathological examination of the resected specimen. Despite receiving radiation and chemotherapy, the patient died 6 months after the first admission. At autopsy, the boundary between the tumor and normal brain tissue was clear. Large parts of the tumor demonstrated typical features of PXA, including pleomorphism, clear xanthomatous cells with foamy cytoplasm, positive silver staining, and a Ki-67 index of less than 1 %.

**Discussion and conclusions:**

GBM should be diagnosed only when the majority of the tumor cells are undifferentiated. Although the operative specimen appeared typical GBM histologically, the diagnosis of GBM was subsequently excluded by the autopsy finding that much of the tumor had the characteristic features of a benign PXA. Therefore, the final diagnosis in this case was PXA with anaplastic features. PXA with anaplastic features should be carefully distinguished from GBM to facilitate appropriate decisions concerning treatment.

## Background

The characteristic histological features of pleomorphic xanthoastrocytoma (PXA) include strong pleomorphism resembling that of glioblastoma multiforme (GBM); however, according to the WHO classification, failure to identify mitosis and necrosis should result in a diagnosis of PXA, which is classified as a grade II glial tumor [1]. The prognosis of PXA is relatively favorable; however, malignant transformation with aggressive clinical behavior has been reported in some recurrences [2, 3]; transformation to GBM has also been described [4]. When anaplastic components are identified in a tumor, the diagnosis is PXA with anaplastic features [5, 6]. We here present a case in which we believe the diagnoses of malignant transformation of PXA or PXA with anaplastic features were both inappropriate, because only the characteristic features of GBM were found on histological examination of the initial specimen, a partially resected tumor, whereas PXA was diagnosed at autopsy, when the entire tumor could be carefully examined.

## Case presentation

**Fig. 1:**
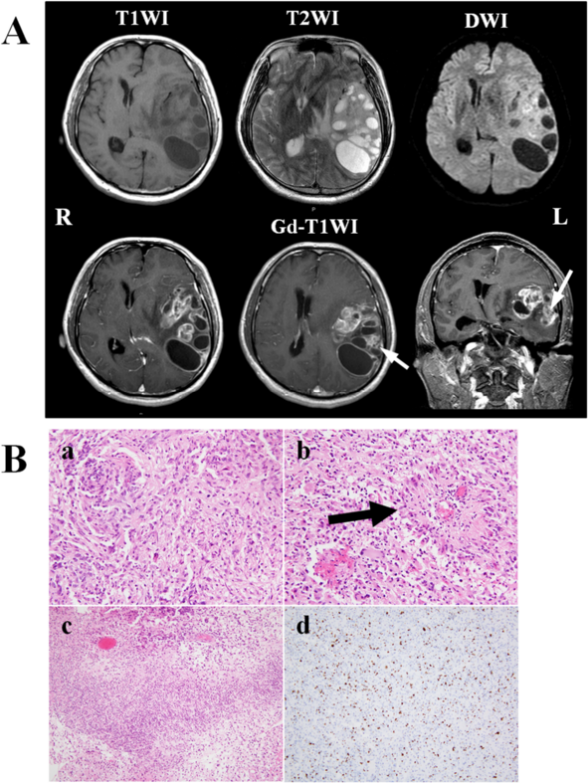
**a** MRI before biopsy. T1-wighted image (T1WI) shows multiple cystic lesions in the left temporal lobe causing a left to right midline shift. These cysts are displayed as high signals in T2-weighted image (T2WI) and low signals in diffusion weighted image (DWI). T2WI also demonstrate edematous brain tissue around the cysts, some parts of which shows high signals in DWI. The cyst walls and surrounding tissue are definitely enhanced by gadolinium dimeglumine (*lower line*). *White arrows* indicate the enhanced portion from which the biopsy was taken. **b** Pathological findings on biopsy. Hematoxylin and eosin (HE) stained section showing high cellularity and dysplasia with endothelial proliferation in the lesion (*a*). Giant cells and pseudo-rosettes are also identifiable (*b black arrow*), and areas of necrosis sections are present (*c*). The highest Ki-67 index is 20 % (*d*)

A 42-year-old man who had been treated for depression for 3 years visited our clinic because of right motor weakness and aphasia that had worsened progressively over 1 month. Manual muscle testing (MMT) revealed right motor weakness of 3/5 in the upper limb and 4/5 in the lower limb and he had total aphasia. Head magnetic resonance imaging (MRI) (Fig. 1A) demonstrated multiple cystic lesions in the left temporal lobe. These cysts had low signals in T1-weighted (T1WI) and diffusion-weighted images (DWI), and high signals in T2WI. The parenchymal components around the cysts showed slightly high signals in DWI. The parenchymal portions and cyst walls were enhanced by gadolinium dimeglumine. A pronounced left to right midline shift caused by the mass effect of the cysts and brain edema was evident. Neuroradiologists commented that the most possible diagnosis was GBM and malignant oligodendroglioma should be a differential diagnosis considering the patient’s age.

The tumor was partially removed and the largest cyst opened under general anesthesia (the white arrows in Fig. 1A indicate the resected portion.). The tumor was yellowish and soft and the fluid in the cyst was weakly xanthochromic. Histological examination of the resected tissue resulted in a diagnosis of GBM (Fig. 1B).

**Fig. 2:**
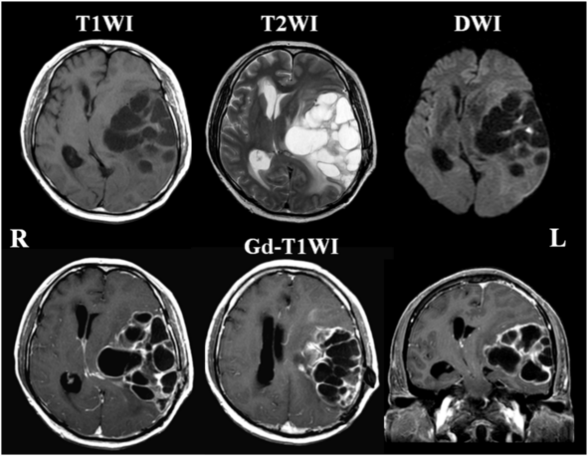
MRI 3 months after treatment The size and number of the cystic lesions have increased with a pronounced left to right midline shift demonstrating impending cerebral herniation. Enhanced lesions still localized surround the cysts and there is no evidence of tumor extension into the basal ganglia or corpus callosum

One month after surgery, radiation of 60 Gy (2 Gy × 30 times) and oral temozolomide (TMZ) treatment (75 mg/m^2^) for 42 days were initiated [7]. After this treatment, the right lower extremity’s motor function recovered to 5/5 on MMT; however, the right upper limb motor weakness and aphasia did not improve with the treatment. One week after completion of irradiation and TMZ treatment, the patient was discharged to home and continued to take TMZ treatment for 5 days every 4 weeks. Three months later, he was brought to our emergency room because of deterioration in consciousness. His Glasgow coma scale was 4 (E1M1M2), and MRI demonstrated enlargement of several of his cysts, causing uncal herniation (Fig. 2A). The patient’s parents refused further treatment on his behalf and he died 5 days after this admission. An autopsy was performed 6 h after his death with the permission of the patient’s parents.

### Pathological findings

#### Pathological findings on biopsy

Histological examination of the operative specimen resulted in a diagnosis of GBM, and this diagnosis was based on the following characteristic features; high cellularity, pleomorphism with giant cells, endothelial proliferation, pseudo-rosette formation, necrosis (Fig. 1B a, b, c), and a high Ki-67 index of 20 % (Fig. 1B d).

#### Histological findings on autopsy

**Fig. 3:**
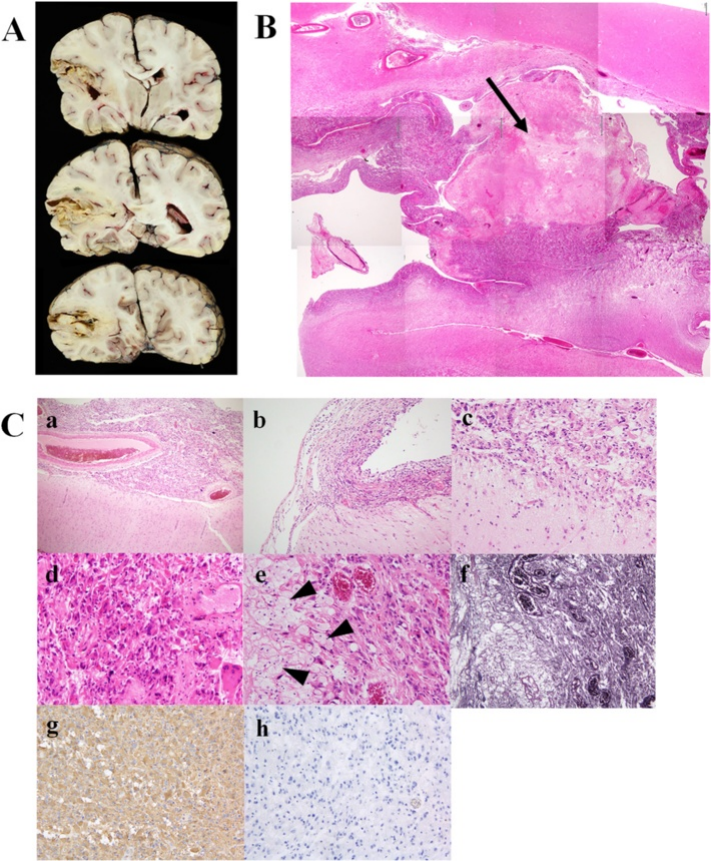
Pathological findings on autopsy. **a** Macroscopic observation of the brain shows tumor tissue only close to the cysts without aggressive invasion of deep white matter. **b** Necrosis is clearly apparent in the tumor (*black arrow*). **c** Higher magnification of the tumor tissue showing it has well-defined boundaries (*a*, *b*, *c*). Pleomorphism of the tumor with giant cells is readily apparent (*d*). Clear xanthomatous cells with foamy cytoplasm are visible (*black arrowheads*) in the pleomorphic portion of the tumor (*e*), and the area is strongly positive on silver staining (*f*). The tumor is strongly positive for glial fibrillary acidic protein (*g*) and the Ki-67 index is extremely low as 1 % (*h*)

On gross morphological examination, the cysts in the tumor shrank after formaldehyde fixation and the parenchymal portion of the tumor was clearly identified. An absence of destructive tumor invasion of the white matter indicated it was not aggressive (Fig. 3A). On low magnification of hematoxylin and eosin (HE) stained sections, tumor necrosis was evident but the boundary between the tumor and normal surrounding tissue was well-defined (Fig. 3B). Examination under higher magnification confirmed well-defined tumor boundaries (Fig. 3C a, b, c). The histologic appearance was heterogenous and pleomorphic giant cells were readily identified (Fig. 3C d). Clear xanthomatous cells with foamy cytoplasm were apparent (Fig. 3C e) and were strongly positive on silver staining (Fig. 3C f). The tumor was strongly glial fibrillary acidic protein positive (Fig. 3C g) and Ki-67 index was extremely low as 1 % (Fig. 3C h).

## Conclusions

Malignant tumors contain areas of different malignant grades. The most malignant grade determines the grade for the entire tumor, even when most of the tumor is of lower grade [8, 9]. According to this rule, our patient’s tumor was a grade IV GBM, which is in accordance with his short survival of 6 months from the first admission.

However, we considered that the diagnosis of GBM is incorrect in our case for the following reasons: (i) the features identified on MRI, in particular the multiple cystic lesions with little intervening parenchyma and minimal invasion to the basal ganglia, are not typical of GBM and (ii) histological examination of autopsy specimens demonstrated typical PXA with well-defined boundaries between the tumor and the surrounding normal brain tissue and the highest Ki-67 index was 1 %. A decrease in median Ki-67 index after chemotherapy has been reported in 106 patients with breast cancer, this index decreasing to 5.21 % from 16.68 % [10]. In our case, the Ki-67 index may have been reduced by irradiation and TMZ treatment; however, a drop of the index to 1 % from 20 % seems too pronounced to be attributable to treatment. Additionally, it seems extremely improbable that a GBM, the most malignant brain tumor, would revert to being a benign tumor with treatment.

In our case, the most crucial finding that is incompatible with a diagnosis of GBM is that typical characteristics of PXA were identified in most parts of the tumor at autopsy. According to the histological definition of grade IV glioma, almost 100 % of these tumors is composed of undifferentiated cells and few well-differentiated cells are present [11]. Hirose et al. have stated that anaplastic PXA should be distinguished from GBM by identifying the salient histological features of conventional PXA even in the anaplastic areas [12]. Therefore, after careful consideration, we conclude the correct histological diagnosis in our case is PXA with anaplastic features, even though GBM was diagnosed on histological examination of the original operative specimen.

## Abbreviations

PXA, pleomorphic xanthoastrocytoma; GBM, glioblastoma multiforme; MMT, manual muscle testing; MRI, magnetic resonance imaging; T1WI, T1-weighted images; DWI, diffusion-weighted images; T2WI, T2-weighted images; TMZ, temozolomide
